# Varicella Virus-Host Interactions During Latency and Reactivation: Lessons From Simian Varicella Virus

**DOI:** 10.3389/fmicb.2018.03170

**Published:** 2018-12-21

**Authors:** Océane Sorel, Ilhem Messaoudi

**Affiliations:** Department of Molecular Biology and Biochemistry, University of California, Irvine, Irvine, CA, United States

**Keywords:** herpesvirus, viral latency, varicella zoster virus, simian varicella virus, non-human primates, viral reactivation, shingles

## Abstract

Varicella zoster virus (VZV) is a neurotropic alphaherpesvirus and the causative agent of varicella (chickenpox) in humans. Following primary infection, VZV establishes latency in the sensory ganglia and can reactivate to cause herpes zoster, more commonly known as shingles, which causes significant morbidity, and on rare occasions mortality, in the elderly. Because VZV infection is highly restricted to humans, the development of a reliable animal model has been challenging, and our understanding of VZV pathogenesis remains incomplete. As an alternative, infection of rhesus macaques with the homologous simian varicella virus (SVV) recapitulates the hallmarks of VZV infection and thus constitutes a robust animal model to provide critical insights into VZV pathogenesis and the host antiviral response. In this model, SVV infection results in the development of varicella during primary infection, generation of an adaptive immune response, establishment of latency in the sensory ganglia, and viral reactivation upon immune suppression. In this review, we discuss our current knowledge about host and viral factors involved in the establishment of SVV latency and reactivation as well as the important role played by T cells in SVV pathogenesis and antiviral immunity.

## Introduction and Knowledge Gaps

Varicella zoster virus (VZV) is one of the nine human herpesviruses. Primary VZV infection results in varicella (also known as chickenpox), a disease characterized by a vesicular rash, fever, headache, and loss of appetite (Heininger and Seward, 2006). Like other alphaherpesviruses, VZV exhibits neurotropism and establishes latency in sensory ganglia neurons. VZV transmission is thought to occur through either inhalation of saliva droplets containing infectious particles and by direct contact with virus in varicella or zoster skin lesions (Leclair et al., 1980; Sawyer et al., 1994; Suzuki et al., 2004). Subsequently, VZV is presumed to undergo initial replication in the upper respiratory tract and tonsillar lymph nodes before viremia and dissemination to the skin leading to the development of varicella (Zerboni et al., 2014). Although primary VZV infection in immunocompetent individuals usually results in a benign disease, serious complications can occur in immune compromised individuals, including pneumonia, secondary bacterial infection, and stroke (Gnann, 2002; Chiner et al., 2010; Wiegering et al., 2011). Two hypotheses are proposed to explain how VZV reaches the ganglia: (Heininger and Seward, 2006) VZV infects sensory neurons via retrograde axonal transport from the infected skin, and (Suzuki et al., 2004) VZV is carried by infected T cells to the ganglia through the hematogenous route (Depledge et al., 2018b). During reactivation, VZV travels from the ganglia to the skin via anterograde axonal transport to cause herpes zoster (HZ, also known as shingles), a painful and debilitating disease that primarily affects the elderly and immunocompromised. HZ is characterized by severe prodromal pain followed by a rash restricted to the dermatome innervated by the ganglia from which the virus reactivated (Wareham and Breuer, 2007). The incidence of HZ is estimated to be 3 per 1000 adults between the age of 40 and 50 years old and increases to 11 cases per 1000 adults above the age of 80 years old (Keating, 2016). VZV reactivation can also cause other complications such as HZ ophthalmicus, vasculitis, stroke, as well as pain without development of a rash, referred as zoster sine herpete (Dayan and Peleg, 2017). Routine vaccination of children against chickenpox was implemented in several countries including Japan (1988), the United States (1995), and Canada (1999) using the live attenuated VZV vaccine that was derived from the Oka strain (Gershon, 2017). There are currently two available vaccines to prevent HZ in the elderly: a live-attenuated (Zostavax^®^, licensed 2005, ∼55% efficacious) and a recombinant (Shingrix^®^, licensed 2018, 97% efficacious) vaccine (Arnold and Messaoudi, 2017a; James et al., 2018).

Despite extensive studies, our understanding of VZV pathogenesis remains incomplete. First, the mechanisms by which VZV disseminates from the initial site of infection to the skin and ganglia are poorly understood. The prevailing model proposes that VZV initially replicates within mucosal epithelial cells at the sites of entry, followed by spread to tonsils and other regional lymphoid tissues, where VZV gains access to T cells that deliver the virus to cutaneous sites of replication and sensory ganglia (Zerboni et al., 2014). However, this model was constructed primarily using data obtained from *in vitro* studies carried out using the attenuated Oka vaccine strain and *in vivo* studies utilizing a severe-combined immunodeficient (SCID) mouse model implanted with human fetal tissues (SCID-hu) (Moffat et al., 1995; Ku et al., 2004). Moreover, the exact timeline as well as the mechanisms through which the latency is established and maintained following primary infection still remains unclear. In order to address these questions, a reliable animal model that recapitulates the key hallmarks of VZV infection is necessary.

## Simian Varicella Virus Infection: an *In Vivo* Model to Study Varicella Zoster Virus Pathogenesis

Numerous attempts have been made to develop a reliable animal model that recapitulates the hallmarks of VZV infection. However, the success of these models remains limited due to the strict human specificity of VZV. Although seroconversion was observed following VZV inoculation in different rodent models including guinea pigs, mice, and rats; no virus was detected in circulation in these models (Haberthur and Messaoudi, 2013). Infection of guinea pigs was rendered possible through the derivation of a guinea pig-adapted VZV strain (by passaging the virus multiple times in fetal guinea pig cells) and injection of peripheral blood mononuclear cells (PBMCs) that are first infected *in vitro* (Gan et al., 2014). Although VZV was shown to establish latency in enteric neurons *in vivo*, the inconsistent development of both viremia and rash in addition to the inability to induce VZV reactivation *in vivo* limits the use of this small animal model (Haberthur and Messaoudi, 2013). Reactivation can be induced *in vitro* through overexpression of VZV ORF61 in latently infected guinea pigs enteric neurons (Gershon et al., 2008). Subcutaneous injection of VZV-infected cells in rats was reported to lead to establishment of a latency-like quiescent state in sensory ganglia although the virus was not shown to be able to reactivate (Annunziato et al., 1998; Sadzot-Delvaux et al., 1990). In addition, footpad inoculation of VZV-infected cells in the rat model has been used to study post-herpetic neuralgia (PHN), long-term chronic pain associated with zoster (Dalziel et al., 2004). Inoculation of non-human primates with VZV also resulted in latency and the development of immunity in the absence of viremia or varicella, suggestive of abortive infection (Felsenfeld and Schmidt, 1979; Meyer et al., 2015a; Myers et al., 1987, Provost et al., 1987; Cohen et al., 1996; Willer et al., 2012). Intradermal inoculation of chimpanzees resulted in a local rash, however, several restrictions have been placed on the use of apes for biomedical research (Myers et al., 1987; Cohen et al., 1996).

In order to overcome the host specificity restriction of VZV, a humanized SCID mouse model was developed using human tissue xenografts. The engraftment of different human fetal tissues (thymus/liver, skin, ganglia, and lung) in this model allowed direct inoculation of VZV and resulted in several important insights into VZV pathogenesis (Moffat et al., 1995; Ku et al., 2004; Zerboni et al., 2005; Reichelt et al., 2008; Wang et al., 2017). However, this model also presents several limitations including: (1) direct inoculation into the human xenografts tissues does not mimic natural route of transmission; (2) the lack of adaptive immunity, which is critical to control viral infection; and (3) the possibility that the strict human host specificity of VZV may alter virus behavior in this model; (4) the use of the attenuated Oka vaccine strain in some of these studies, which compared to the parent wild type strain contains numerous nucleotide substitutions found in multiple open reading frames (ORFs) and may therefore not accurately model the behavior of wild type virus strains (Jones and Arvin, 2003; Yamanishi, 2008; Sen et al., 2015).

To overcome these limitations an alternative animal model was developed where non-human primates are inoculated with Simian varicella virus (SVV), an alphaherpesvirus that causes a vesicular rash in Old World monkeys. SVV and VZV virions have a diameter of 170–200 nm and 80–120 nm, respectively, and are composed of a nucleocapsid of icosahedral symmetry surrounded by a viral envelope (Gray, 2010). The nucleocapsid of both SVV and VZV contains a linear double-stranded DNA genome of 124,138 and 124,884 bp, respectively. The viral genomes of SVV and VZV include a unique long sequence of 104.1 and 104.8 kb, respectively, and a unique short region that comprises a 4.9 and 5.2 kb sequence for SVV and VZV, respectively, as well as internal repeat and terminal repeat regions (Clarke et al., 1992). SVV and VZV genomes share 70–75% DNA homology (Gray and Oakes, 1984) and an amino acid identity ranging from 27 to 75% (Gray et al., 2001). Both VZV and SVV encode 74 ORFs of which 71 are distinct and 3 (ORFs 69, 70, and 71) are duplicated within the repeat regions (Mahalingam and Gilden, 2007; Zerboni et al., 2014). Despite exhibiting co-linearity with respect to gene organization, SVV ORFA is absent in VZV genome while SVV does not include a gene homolog of VZV ORF2 (Gray et al., 2001).

**FIGURE 1:**
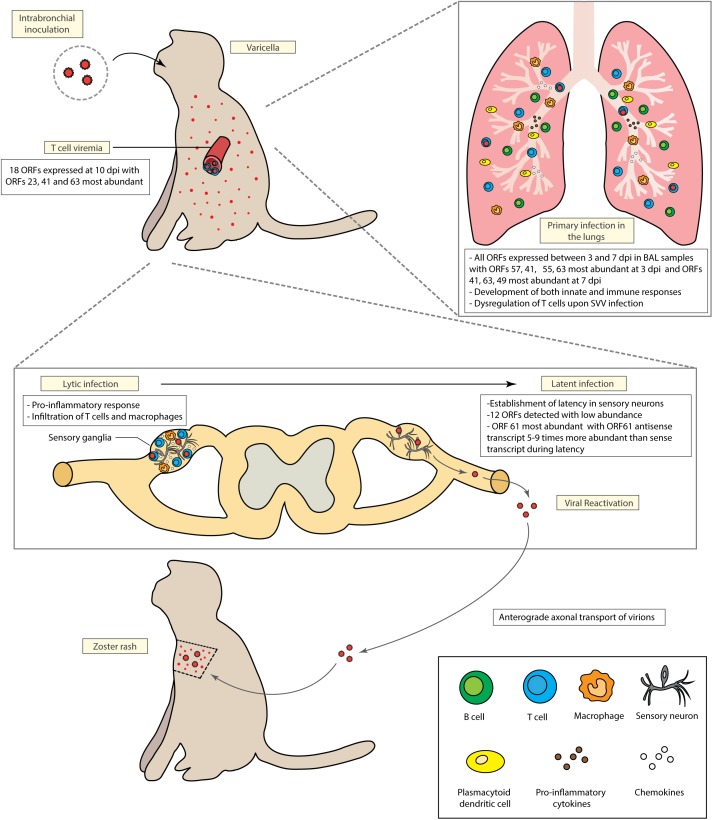
Model of Simian Varicella Virus (SVV) pathogenesis in rhesus macaques following intrabronchial inoculation. Intrabronchial inoculation of rhesus macaques with SVV results in primary infection associated with viral replication in the lung, T cell viremia and the development of varicella. SVV reaches sensory ganglia as early as 3 days post infection. Initial viral replication is followed by the establishment of latency in the sensory ganglia. SVV can reactivate upon immune suppression. Both primary infection and viral reactivation induce robust cellular and humoral immune responses.

The first outbreak of a varicella-like disease in non-human primates was reported in 1967 followed by several epizootics in primates facilities worldwide (Clarkson et al., 1967; Gray, 2008). Depending on the non-human species, SVV infection can cause disease that ranges from a mild varicella (in rhesus macaques also called *Macaca mulata*) to a severe and life-threatening disease associated with high morbidity and mortality rates [cynomolgus monkeys (*Macaca fascicularis*) and African green monkeys (*Chlorocebus sabaeus*)] (Gray, 2008). This spectrum of disease outcomes is a hallmark of herpesvirus infection in species that are closely related to the natural host, e.g., Macacine herpesvirus 1 (also known as herpes simian B virus) infection in humans (Elmore and Eberle, 2008), Elephant endotheliotropic herpesvirus infection in Asian elephants (Long et al., 2016), and Alcelaphine herpesvirus 1 in cattle (Sorel et al., 2017). Moreover, SVV infection in cynomolgus monkeys and African green monkeys results in persistent viremia which limits the use of these models to study the adaptive immune response against SVV (White et al., 2002; Mahalingam et al., 2007). In contrast, intra-bronchial inoculation of rhesus macaques with SVV faithfully recapitulates the hallmarks of VZV pathogenesis including: viremia, development of varicella, generation of robust cellular and humoral immune responses, establishment of latency in the sensory ganglia, and viral reactivation following immune suppression (Messaoudi et al., 2009; Meyer et al., 2011; Haberthur et al., 2013, 2014; Arnold et al., 2017; Figure 1).

## Role of T Cells in SVV Trafficking

Data from several studies carried out using the hu-SCID mouse model strongly suggest a critical role for T cells in VZV dissemination. First, direct inoculation of VZV-infected fibroblasts into human fetal thymus and liver xenografts placed under the kidney capsule of SCID mice revealed that T cells support VZV replication (Moffat et al., 1995). Furthermore, injection of VZV-infected T cells into human skin xenografts implanted in the SCID model demonstrated the importance of the type I interferon response in restricting VZV replication in the skin (Ku et al., 2004). More importantly, intravenous injection of VZV-infected T cells but not fibroblasts resulted in vesicular rash of human skin implants suggesting that T cells can traffic the virus to the skin (Ku et al., 2004). These observations gave rise to the current model which stipulates that VZV gains access to T cells that deliver the virus to cutaneous sites of replication (Zerboni et al., 2014). However, given the limitations of this mouse model, findings from these studies cannot be extrapolated to decisively uncover the mechanisms by which VZV hijacks T cells to disseminate to the ganglia in the human host *in vivo*.

In both rhesus macaques and African green monkeys, different subsets of immune cells, including T cells, were shown to reach the ganglia as early as 3 days post intrabronchial inoculation (dpi) during acute infection (Ouwendijk et al., 2013b; Arnold et al., 2016a), prior to the detection of anti-SVV specific T cell immunity, these results suggest that T cells play an important role in SVV dissemination to sensory ganglia. Interestingly, CD8 memory T cells were the most abundant subset of immune cells infiltrating the ganglia of SVV-infected African green monkeys and rhesus macaques (Arnold et al., 2016a; Ouwendijk et al., 2016). In support of the potential role of T cells as Trojan horse for SVV, T cells isolated from bronchial alveolar lavage (BAL) samples during primary infection supported viral replication (Arnold et al., 2016a). Similarly, T cells infiltrate the enteric nervous system during acute SVV infection in African green monkeys (Ouwendijk et al., 2018). These results obtained in the SVV infection model along with the studies reporting that VZV infected T cells can traffic the virus to human xenografts in the SCID-hu mouse model further emphasize the importance played by T cells in varicella viruses dissemination and pathogenesis (Ku et al., 2004; Zerboni et al., 2005).

In order to improve our understanding of the mechanisms by which SVV alters T cells migratory behavior and to in SVV trafficking, a recent study analyzed the transcriptional profile of T cells in BAL samples following SVV infection in rhesus macaques (Arnold and Messaoudi, 2017b). This study reported that multiple cellular processes were dysregulated in T cells upon SVV infection, including genes involved in chromatin assembly, immune response, cell cycle, and cellular metabolism. These results suggest that SVV might alter T cell functions in order to achieve efficient viral replication and allow the virus to spread in the host while evading the immune system. Interestingly, in line with this hypothesis, previous *in vitro* studies using single-cell mass-spectrometry analysis (CyTOF) have reported that VZV infection of human tonsil T cells leads to upregulation of several immune genes including components of the TCR signaling machinery (Sen et al., 2014, 2015).

## SVV Latency Pattern in the Rhesus Macaque Model

Transcriptomic analysis of BAL cells collected from rhesus macaques during SVV primary infection showed that all ORFs were expressed between 3 and 7 dpi with increasing intensity that correlated with viremia (Meyer et al., 2011). The most highly expressed SVV ORFs detected at 3 dpi in BAL samples were ORF 57 (unknown function), ORF 41 (capsid protein), ORF 55 (component of the DNA helicase-primase complex), and ORF 63 (transactional activator) (Meyer et al., 2011). At 7 dpi, ORF63, ORF41 as well as ORF49 (structural protein) were the most abundant transcripts expressed in BAL cells (Meyer et al., 2011). Parallel analysis of the viral transcriptome profiles of PBMCs derived from rhesus macaques infected with SVV revealed that only 18 SVV ORFs were expressed at 10 dpi in these samples, consistent with significantly lower viremia (Meyer et al., 2011). However, similarly to the BAL samples, the most highly expressed viral genes included ORFs 23, 41, and 63 (Meyer et al., 2011).

Latency is a state that is characterized by a restricted viral gene expression pattern. In line with that expectation, only 12 SVV ORFs were detected sporadically during SVV latent infection in the sensory ganglia. Importantly, ORF61 was the most abundant and consistently detected transcript in the ganglia during latent infection (Meyer et al., 2011). More specifically, the antisense transcript of ORF61 was found to be 5–9 times more abundant than sense transcripts (Messaoudi et al., 2009). Similar findings were reported for latently infected ganglia collected from Vervet and African green monkeys (Ou et al., 2007; Ouwendijk et al., 2013a). Recently, the presence of an ORF61 anti-sense transcript was reported for VZV latent infection (Depledge et al., 2018a). Although multiple isoforms of VZV ORF61 antisense transcripts were detected during lytic infection, only one isoform was predominant during latency and was shown to suppress VZV ORF61 expression (Depledge et al., 2018a). Taken together, these results suggest that cessation of ORF61 expression by the anti-sense transcript may be critical in the establishment and maintenance of latency. Interestingly, establishment of latency was not impaired in animals infected with an SVV mutant deleted of ORF61 (SVVΔORF61) (Meyer et al., 2013c). Since ORF61 is thought to be shut off by the anti-sense transcript in order to prevent reactivation during latency, the lack of the ORF61 anti-sense transcript following infection with SVVΔORF61 would explain why establishment and maintenance of latency are not affected by the deletion.

SVV ORF61 is an immediate early gene that encodes a protein with a RING finger motif at the amino terminus, which is important for potential E3 ubiquitin ligase activity as well as a nuclear localization signal at the N terminus (Gray et al., 2007). Previous *in vitro* studies showed that ORF61 protein can transactivate its own promoter as well as promoters of SVV genes of all kinetic classes (Gray et al., 2007). Although SVV ORF61 is non-essential for SVV lytic cycle *in vitro*, SVVΔORF61 replicates 2- to 5-fold less efficiently compared to the wild-type (WT) virus (Gray et al., 2007). Similarly, *in vivo* infection with SVVΔORF61 was associated to a decreased expression of all viral transcripts and decreased viral loads in rhesus macaques (Meyer et al., 2013c). Infection with SVVΔORF61 also led to increased infiltration of plasmacytoid dendritic cells (pDC) into the lungs and expression of interferon stimulated genes *in vivo* suggesting a potential role of ORF61 in evasion of the host innate immune response (Meyer et al., 2013c). Indeed, both SVV and VZV ORF61 were shown to interfere with NF-κB signaling *in vitro* (Whitmer et al., 2015).

## The Important Role of T Cells in the Establishment and Maintenance of Latency

Intrabronchial infection of rhesus macaques with SVV results in the development of both innate and adaptive immune responses in the lungs concomitant with a decrease in the SVV viral loads observed (Arnold et al., 2016b). The mucosal innate immune response is characterized by a significant production of pro-inflammatory cytokines, chemokines (including T cell chemoattractants) and IFNα into the alveolar space that correlates with increased frequency of pDCs (Haberthur et al., 2014; Arnold et al., 2016b). This initial response is followed by a robust proliferation and infiltration of B and T cells in the lungs (Haberthur et al., 2014). Although CD8 T cells were found to be more abundant, a higher proportion of CD4 T cells were specific to SVV in the BAL (Haberthur et al., 2014). This observation is in line with several studies that reported a critical role for CD4 T cells in controlling both SVV and VZV acute infection (Haberthur et al., 2011; Duncan and Hambleton, 2015; Sen et al., 2015; Sen and Arvin, 2016). Indeed, whereas depletion of B cells and CD8 T cells showed no or limited effect on disease severity, CD4 depletion led to higher viral loads, prolonged viremia, and disseminated varicella (Haberthur et al., 2011). These results explain why children with T cell deficiencies are more prone to developing serious complications following VZV infection whereas children with B cell deficiencies have uncomplicated disease (Arvin et al., 1978; Wilson et al., 1992; Nader et al., 1995; Redman et al., 1997; Zerboni et al., 1998).

The anti-SVV T cell responses during acute infection in rhesus macaques is broad with CD8 T cell responses directed mainly against immediate-early (IE) and early (E) viral proteins whereas CD4 T cell responses were mostly specific to late (L) proteins (Haberthur et al., 2013). During latency, the magnitude of the T cell response decreases dramatically and becomes more restricted (Haberthur et al., 2013). Specifically, T cell responses directed against only 5 ORFs (ORF 4, 11, 19, 31, and 37) were maintained during latency whereas specific T cell responses to ORFs 10, 20, 29, 31, 62, 63, 68 showed a significant decrease compared to primary infection (Haberthur et al., 2013). Amongst these viral antigens, ORF68 (gE) is the most abundant glycoprotein, a critical determinant of VZV pathogenesis (Moffat et al., 2004; Berarducci et al., 2009; Zerboni et al., 2011), and a highly immunogenic viral antigen (Vizoso Pinto et al., 2010). These data suggest that boosting T cell responses against these viral antigens that are highly immunogenic during acute infection but poorly recognized during latency may be a promising direction for HZ vaccine. Indeed, the highly efficacious new recombinant subunit HZ vaccine (Shingrix^®^) contains an adjuvanted form of VZV gE that was shown to elicit a robust humoral and cell-mediated immunity (Mo et al., 2002; James et al., 2018; Syed, 2018). In contrast, Zostavax^TM^ induces a lower VZV-specific cell-mediated immunity including a reduced gE-specific memory T cell responses compared to Shingrix^®^(Levin et al., 2018; Weinberg et al., 2018).

The importance of T cell responses during acute infection in the establishment of latency is evidenced by the detection of high level of viral transcription in ganglia of animals depleted of CD4 T cells during acute infection (Meyer et al., 2013b). It should be noted that at the time of ganglia analysis, the animals were no longer viremic. These results strongly suggest that loss of CD4 T cell immunity during acute infection impaired the establishment of a latency in sensory ganglia of infected macaques (Meyer et al., 2013b). In accordance with this observation, SVV infection of aged rhesus macaques was also characterized by dampened T cell responses and high levels of viral transcription inconsistent with latent infection (Meyer et al., 2013b). More recently, direct inoculation of VZV-infected fibroblasts into human fetal dorsal root ganglia (DRG) implanted under the kidney capsule as well as intravenous transfer of VZV-infected CD4 T cells showed persistent viral replication in the ganglia tissue followed eventually by latency (Zerboni et al., 2005; Reichelt et al., 2008). These data from the hu-SCID mouse suggest that adaptive immune responses may not be critical for the establishment of latency. However, the CD4 T cells were most likely obtained from VZV-seropositive individuals and therefore the fact that they may harbor VZV-specific T cells cannot be dismissed. Similarly to VZV, stress and immune suppression can induce SVV reactivation leading to anterograde axonal transport of virions to the skin causing HZ lesions (Soike et al., 1984; Mahalingam et al., 2007, 2010; Traina-Dorge et al., 2014). Because T cells were shown to be critical in the establishment of latency, a recent study investigated the specific role of T cell immunity in preventing SVV reactivation (Arnold et al., 2017). This study showed that depletion of either CD4 or CD8 T cells in latently infected animals led to subclinical reactivation (defined as viremia detected in the absence of zoster rash) and an increase in the viral loads in the ganglia (Arnold et al., 2017). Moreover, large transcriptional changes of genes involved in inflammation and neuronal functions were reported in the ganglia obtained from animals that experienced subclinical reactivation (Arnold et al., 2017). Taken together, these results support the critical role of T cell immunity in maintaining SVV latency.

## Immunological Outcomes Following Reactivation

Other studies have attempted to induce reactivation in SVV-latently infected rhesus macaques using a combination of total body irradiation (2–8 Gy) and immune suppressant regimens (cyclosporine and tacrolimus). In some of these studies, cynomolgus and rhesus macaques were irradiated before receiving tacrolimus and prednisone, resulting in clinical reactivation in 25 and 100% of animals, respectively (Mahalingam et al., 2007; Traina-Dorge et al., 2014). In another study, treatment with only immune suppressants resulted in 75% reactivation (Ouwendijk et al., 2013a). The incidence of HZ obtained following these experimental treatments is significantly higher than the reactivation rate reported in humans. Another perplexing outcome of these studies includes the very high incidence of reactivation in the non-treated controls which is often ∼100%. Other groups failed to reproduce these findings using the same approaches (Meyer et al., 2015b), potentially due to a higher level of stress induced by a longer transportation to the irradiation site for the animals in the compared to those housed in the Oregon National Primate Center.

Following reactivation, SVV antigens were detected in multiple tissues, including skin and lymph nodes in rhesus macaques despite the lack of viremia at the time of HZ (Traina-Dorge et al., 2015). In skin tissues, SVV antigens were found mainly in sweat glands, whereas in lymph nodes, they were detected in macrophages, dendritic cells (DCs), and T cells. It is possible that DCs containing SVV antigens are activating T cells in the peripheral lymph nodes or that infected DCs are transferring SVV to T cells as previously described for VZV (Abendroth et al., 2001). Additionally, SVV reactivation in rhesus macaques induces the development of a strong systemic pro-inflammatory response (Traina-Dorge et al., 2014) associated with an overall increased in the number of total T cells compared to latency (James et al., 2014). T cell infiltration was detected in the sensory ganglia of cynomolgus macaques experiencing reactivation where neurons were found to be surrounded mainly by CD8 rather than CD4 T cells (Ouwendijk et al., 2013a). Moreover, as previously reported for post-mortem human sensory ganglia derived from patients who suffered from HZ at the time of death (Steain et al., 2011), the authors detected elevated levels of CXCL10, a chemokine involved in T cell migration (Ouwendijk et al., 2013a). Taken together these results suggest that the pro-inflammatory response play an important role in initiating T cell recruitment to the site of SVV/VZV reactivation. However, the high reactivation rates in the control animals raises concerns about the clinical significance of these findings.

## Conclusion and Future Perspectives

Although studies over the last few decades have led to significant advances in our understanding of VZV pathogenesis, several questions remain unanswered. Specifically, although it is now well established that that T cells play a critical in the pathogenesis of both VZV and SVV, the exact mechanisms by which VZV/SVV modulate T cell functions to alter their migratory properties and confer ability to access into the central nervous system are not known. Furthermore, the viral and cellular factors that control establishment and maintenance of SVV/VZV latency in sensory ganglia remain poorly understood. Notably, the role of the ORF61 anti-sense transcript during the transition from lytic to latent phases has yet to be investigated. Similarly, the role of epigenetic modifications (such as histone/DNA methylation or histone acetylation) in the maintenance of latency remains to be studied. Future studies should uncover the impact of persistent transcriptional changes within the ganglia on neuronal function. SVV infection in rhesus macaques provides a model well-suited to further our knowledge of varicella viruses’ pathogenesis. The availability of this model together with a versatile bacterial artificial chromosome (Gray et al., 2011; Meyer et al., 2013a) that facilitates manipulation of the viral genome will play a critical role in addressing these remaining gaps in our knowledge.

## Author Contributions

OS and IM wrote the manuscript.

## Conflict of Interest Statement

The authors declare that the research was conducted in the absence of any commercial or financial relationships that could be construed as a potential conflict of interest.
